# Evaluating burnout syndrome among medical students at the Aristotle University of Thessaloniki in Greece: a cross-sectional study

**DOI:** 10.3389/fpsyt.2025.1538393

**Published:** 2025-05-28

**Authors:** Konstantinos Angelopoulos, Georgia-Nektaria Porfyri, Angelos Ntikos, Ioanna Chioti, Chrysovalantis Fagogenis, Vasiliki Tarantili, Anastasia Konsta

**Affiliations:** ^1^ Primary Medical Service, Medical Corps, Hellenic Army, Symi, Greece; ^2^ Global Health - Disaster Medicine, School of Medicine, National and Kapodistrian University of Athens, Athens, Greece; ^3^ Medical Corps, Military School of Combat Support Officers, Thessaloniki, Greece; ^4^ Faculty of Medicine, Aristotle University of Thessaloniki, Thessaloniki, Greece; ^5^ Graduate of Statistical and Insurance Science, University of Piraeus, Athens, Greece; ^6^ Health Institutions and Health Policies, University of Peloponnese, Corinth, Greece; ^7^ 1^st^Psychiatric Clinic, Papageorgiou Hospital, Aristotle University of Thessaloniki, Thessaloniki, Greece

**Keywords:** burnout syndrome, medical students, mental health, Maslach basic inventory, Greece

## Abstract

**Introduction:**

Burnout syndrome was first evaluated in the working environment of pilots and air-traffic controllers in 1973 and was officially described in scientific terms by the psychologist Christina Maslach. Recent research proves that the syndrome is currently present among healthcare professionals worldwide. Thus, we investigated whether it is also present in the medical student community, which faces the main characteristics of the syndrome from the beginning of their educational career.

**Materials and methods:**

The research was conducted on a sample of 400 Greek medical students at Aristotle University of Thessaloniki, using the Maslach Basic Inventory questionnaire with three sections: exhaustion, depersonalization, and low satisfaction with personal achievements. The sample was analyzed based on the parameters of gender and the year of study of each individual participant.

**Results:**

In total, 33.5% of the participants were found to have a high risk or tendency for burnout syndrome, 11.75% of whom were at high risk and suffered from the syndrome. Moreover, 21.75% of the participants had a tendency toward suffering the syndrome. No remarkable correlation was discovered for the gender variable, whereas, for the year of study, there was an important correlation between the more senior years (5^th^ and 6^th^) and higher risk.

**Discussion:**

The syndrome’s prevalence from the survey is scientifically important, urging the academic community to examine whether, instead of shaping healthy doctors, in reality, the education system produces patients with the syndrome. Protective measures include cognitive-behavioral therapy, mindfulness, the six-stage adult learning technique, periodical screening of the syndrome, as well as encouragement for higher personal achievements.

## 1 Introduction

Burnout syndrome, first introduced by psychologist Herbert Freudenberger in 1974 (1), is currently a constantly rising phenomenon that tends to appear in the occupational environment (2) and is worrying healthcare professionals. Although many definitions are under evaluation to describe the term, the widely accepted one seems to be that provided by the WHO and the psychologist Christina Maslach (1, 3). The definition of the syndrome is conceptualized using three basic features of human behavior: chronic fatigue, cynicism including reduced commitment over social interacting, and no satisfaction for personal achievements. The main cause of all these in the definition is chronic workplace stress that has not been successfully managed. Despite this, the syndrome, despite being classified in the ICD-11 inventory, is still not identified as a mental health disorder (4).

One of the first investigations into workplace burnout was carried out in 1973 in air-traffic controllers after many accidents happened that were attributed to human failure or because of inadequate training, automation monotony, and insufficient equipment. This was the first occupational group to mention vocational “burn out”, a type of fatigue that resulted in a deterioration of both the quantity and quality of produced work. Furthermore, there was an increased occurrence of hypertension and indications of mental health problems. The researchers concluded that those who feared burnout were the most qualified employees, while at the same time, burnout concerns, once set in motion, tended to become a self-fulfilling prophecy. In addition, burnout was proven not to be simply a failure of psychological resilience since most air traffic controllers had experienced military service and had faced extremely demanding conditions (5). Finally, the following paradox was highlighted: professionals who endeavor the hardest to accomplish internal and external professional goals could intensify their risk of burnout, which then contributes to the failure of these professional goals.

Moreover, Maslach, as the main ambassador of the syndrome’s terminology, expanded on the disease by investigating how workplace emotions relate to health. Early studies suggested that the clinical symptoms of burnout syndrome were tied to the mental health and social dynamics of both caregivers and recipients in professional settings (6). Oakley also argued that burnout among healthcare personnel could be the result of this particular continuous pressure for altruism (7). It is evident that air-traffic controllers and doctors share many common occupational factors, such as a timetable, long night shifts, and being alert in order to face emergency situations. A recent meta-analysis proved that among staff working in the ICU, where critical changes in vital signs require emergency help and attendance, 40% suffered from the syndrome (8). Indeed, in Greece, as the most recent available literature on healthcare professionals suggests, after the COVID-19 pandemic, nurses experienced high levels of burnout (9). In this context, a more compact meta-analysis, which was also conducted after the pandemic, provided compelling evidence that physician burnout is associated with the poor function and sustainability of healthcare organizations, primarily by contributing to career disengagement and turnover of physicians and secondarily by reducing the quality of patient care (10).

Focusing on future medical staff, medical students are a population of trainees who face psychological stress early in their studies due to patients’ deaths, life-threatening situations, and cynicism in their steps toward clinical practice, which are all factors that correspond to the definition of the syndrome (11). Taking this into account, with medical training going beyond borders and medical students around the world sharing much in common, more and more research proves that the syndrome has become a major concern in the scientific student community too, especially when we observe the parameter of the duration of studies (12, 13). Thus, it was an interesting question whether burnout syndrome is already present among medical students in Greece, as there was no study available on the topic that we were aware of.

## 2 Materials and methods

### 2.1 Study design

This was a cross-sectional study in which 400 undergraduate medical students from Aristotle University of Thessaloniki, Greece participated. Notably, the undergraduate degree is 6 year, with the initial 2 years principally focused on basic sciences, while contact with clinical experience begins already from the 1^st^ semester, introducing students to primary healthcare and to clinical practice in 3^rd^ year (14).

The study was conducted from 17 to 22 September 2022, when lockdown was finally over and educational activities were back to normal. Medical students were invited to participate in the research voluntarily and anonymously, having provided informed consent during the initial briefing for the survey. Considering that an online survey can provide a large amount of data within a short period, the questionnaires were provided through electronic devices in an online form. The inclusion criteria were i) acceptance to participate, ii) being a medical student from Aristotle University of Thessaloniki, iii) completion of over 96% of survey questions, a setting enabled while creating the online survey to obtain adequately filled out surveys. Out of the 400 responded surveys obtained, none were excluded due to not fulfilling the criterion of 96% completion. Ethical approval was received by the scientific committee provided by the board of the 1^st^ Psychiatric Medical Clinic at Aristotle University of Thessaloniki.

The evaluation of burnout syndrome involves a structured approach using psychometric variables from a scale developed by Maslach and Jackson (15), known as the “Maslach Burnout Inventory” (MBI). In our survey, the edition of the MBI that was selected among many adaptations (16) was the general questionnaire (17), which has been carried out in the Greek language (18).

Its diagnostic value comes from a self-report scale consisting of statements that reflect feelings and attitudes related to everyday work habits or behaviors, measuring three key dimensions: emotional exhaustion, depersonalization, and a lack of personal accomplishment. Each item in the questionnaire measures the frequency of occurrence of everyday behaviors and feelings of the participants. The minimum score for each item was 0 points with an answer of “Never” and a maximum score of 6 points for “Every day”, so scores varied from 0 to 6 points for each item. High-level burnout is demonstrated in the exhaustion section by more than 30 points, in depersonalization by more than 12 points, and in personal achievement by less than 33 points. This multidimensional model proposed by Maslach and Jackson is the most frequently referenced framework in burnout syndrome research (15, 16, 19). Since our purpose was to highlight patients at high risk for the syndrome, the three different aspects of the test were evaluated as a whole by summing up the total score in every category. As explained by the newest ethical and accurate approach edited by Christina Maslach (20), the “overextended” (high score in exhaustion only), “ineffective” (high score in achievements only), and disengaged (high score in depersonalization only) categories were not included as they do not provide a clear image of the overall high-risk patients. Individuals who demonstrated high risk in two categories were captured in this research, as we considered they could provide important arithmetic data for further discussion and evaluation of patients’ future symptoms and behaviour.

### 2.2 Statistical analysis

The study investigated the association between the level of burnout and two main variables, which reflect the most basic and objective features of the population tested. These were gender (nominal variable: female or male) and the year of study (ordinal variable with seven categories: from 1^st^ to 6^th^ year and beyond), which were evaluated in two analyses respectively. The first analysis investigates the relationship between burnout, and the year of studies in order to examine whether the more the students proceed to the next year demanding curriculum, the more they are in risk for burnout. Several statistical tests were employed to assess the strength and significance of this correlation between two ordinal variables, such as Chi-square (χ^2^), after a residual analysis and specifically its linear-by-linear association. Moreover, the gamma coefficient and Somers’ D were also used to assess the strength and variation of the two variables, considering they were both ordinal. The second analysis examined the relationship between burnout and gender, aiming to explore whether burnout levels differ significantly by biological gender in the two different groups of high-risk and non-high-risk students with the use of the Chi-square and the non-parametric Mann**–**Whitney U statistical tests. An alpha error of 5% (p<0.05) was considered the statistical significance threshold for all analyses. The statistical analyses were performed using SPSS (version 30, IBM SPSS Statistics, Armonk, New York, United States).

## 3 Results

### 3.1 Sample characteristics

According to the 400 answers received from the participants, 146 (36.5%) were males and 254 (63.5%) were females. Regarding the year of study, a balanced distribution of participating individuals was achieved (16.5% in 1^st^ year, 16.75% in 2^nd^, 18% in 3^rd^, 15.25% in 4^th^, 18% in 5^th^, 13.75% in 6^th^, and 1.75% students pending graduation).

### 3.2 Burnout distribution

**Figure 1:**
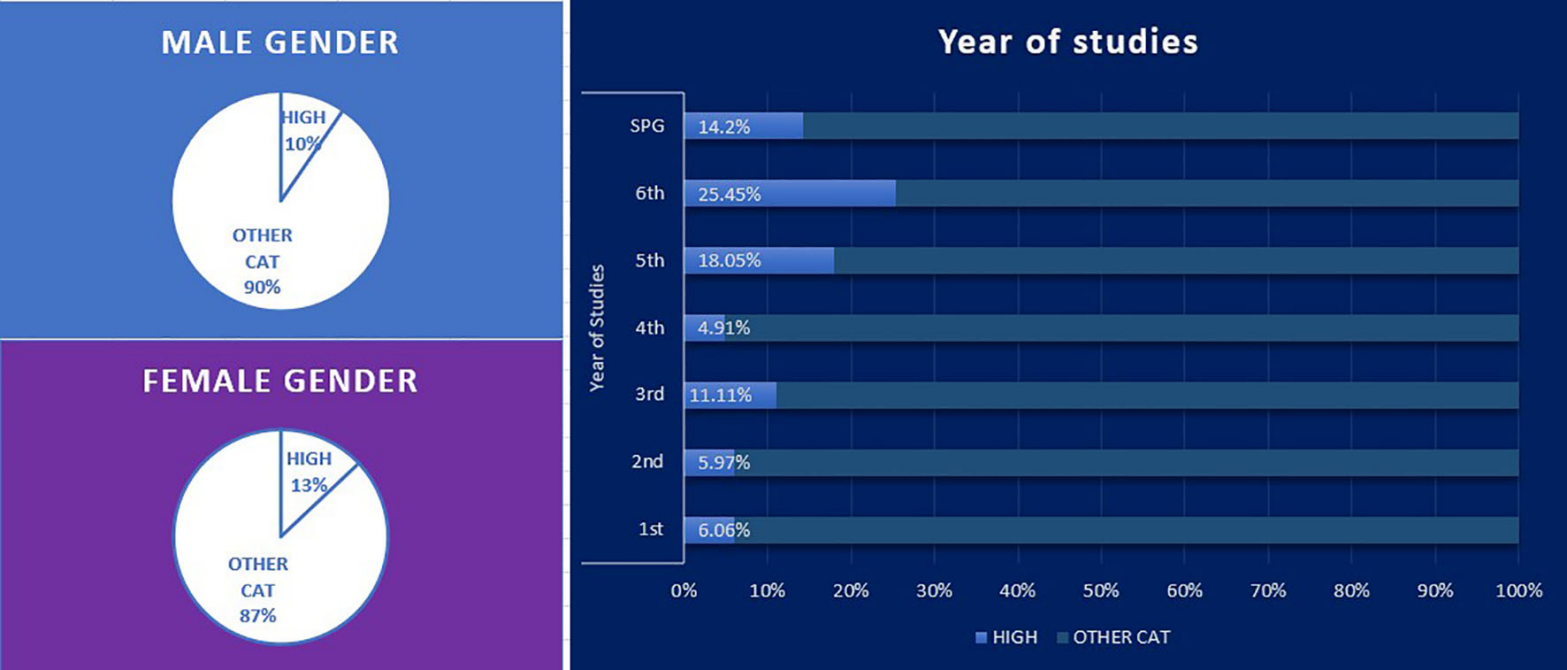
High-risk Students (high risk score in the three parameters of the questionnaire) by gender and by year of study.

The total burnout distribution and total scores highlighted that 11.75% of the total students were at high risk for burnout (20). By gender, 9.59% and 11.99% of male and female students had a high-risk score, respectively. By year of study, the highest percentages of at-risk students were among students in their 6^th^ and 5^th^ years of study, with 1 in 4 of those in their 6^th^ year at high risk (**Figure 1**).

An interesting result was also that 21.75% of participants were at high risk at least in two of the three categories of the questionnaire, with medium risk in the third. This percentage is shown in **Table 1** as “Tendency to”. Even though it is not included in the inventory interpretation, this showed an important rise among participants that should be taken into consideration (20).

**Table 1:** Total burnout distribution by gender and by year of study.

		High	Tendency to	Low or none	Total
**Gender**	Male	14	33	99	**146**
	*Males out of males*	***9.59%***	*22.6%*	*67.81%*	***100%***
	Female	33	54	167	**254**
	*Females out of females*	***12.99%***	*21.25%*	*65.76%*	***100%***
	**Total**	**47 (11.75%)**	**87 (21.75%)**	**266 (66.5%)**	**400 (100%)**

		High	Tendency to	Low or None	Total
**Year of study**	1st	4**(6.06%)**	13(19.7%)	49(72.24%)	**66**
	2nd	4**(5.97%)**	20(29.85%)	43(64.18%)	**67**
	3rd	8**(11.11%)**	13(15.28%)	51(73.61%)	**72**
	4th	3**(4.91%)**	14(22.95%)	44(72.14%)	**61**
	5th	13**(18.05%)**	17(23.6%)	42(58.35%)	**72**
	6th	14**(25.45%)**	10(18.18%)	31(56.37%)	**55**
	SPG^a^	1**(14.2%)**	0(0.0%)	6(85.8%)	**7**
	**Total**	**47(11.75%)**	**87(21.75%)**	**266(66.5%)**	**400(100%)**

a. Students pending graduation (Undergraduate students that exceeded the basic curriculum duration of 6 years).

Finally, 66.5% of the participants showed low or no risk for burnout (**Table 1**). Thus, as a general deduction, 1 in 10 students were at high risk for burnout syndrome.

### 3.3 Statistical analysis of burnout and year of study

#### 3.3.1 Chi-square test

A chi-square test was performed to evaluate the association between burnout and year of study. The resulting p-value of 0.014 indicates a statistically significant relationship between the two variables. Since this p-value is less than the commonly used threshold of 0.05, the null hypothesis was rejected, which assumes no association. This finding suggested that levels of burnout vary significantly across different years of study, indicating that students in higher years may experience different levels of burnout compared to first-year students.

#### 3.3.2 Linear-by-linear association

Since the chi-square test assesses the overall association between categorical variables, we also employed the linear-by-linear association test, a specific form of chi-square test that examines whether there is a statistically significant trend in the relationship between burnout and year of study. This test yielded a p-value of 0.005 and a test statistic of 8.031, indicating a strong and statistically significant linear relationship between the two variables. This suggested that as students progress through their years of study, there was a systematic change in burnout levels, either increasing or decreasing in a predictable manner (**Table 2**).

**Table 2:** Chi-square tests and linear-by-linear association results by the year of study and gender variables.

Chi-Square tests			
Year of study			
	Value	df	Asymptotic Significance (2-sided)
Pearson chi-square	25.142^a^	12	0.014
Likelihood ratio	25.447	12	0.013
**Linear-by-linear association**	8.031	1	0.005
No. of valid cases	400		
a. Three cells (14.3%) have expected count less than 5. The minimum expected count is 0.82			
Gender			
Pearson chi-square	1.050^b^	2	0.592
Likelihood ratio	1.077	2	0.584
No. of valid cases	400		
b. 0 cells (0.0%) have expected count less than 5. The minimum expected count is 17.15.			

#### 3.3.3 Gamma coefficient

The gamma statistic, which assesses the strength and direction of the association between two ordinal variables, produced a value of 0.156 with a p-value of 0.020. The positive gamma value indicates a weak positive association, meaning that higher years of study may be linked to higher levels of burnout. However, the relatively small gamma value suggested that while the association was significant, it was not particularly strong.

#### 3.3.4 Somers’ D

Somers’ D, another measure of association between ordinal variables, yielded a value of 0.098 with a p-value of 0.020. This result similarly indicates a weak but statistically significant positive association between burnout and year of study. This suggested that students in higher years of study may experienced slightly higher levels of burnout, but again, the relationship was not particularly robust.

### 3.4 Statistical analysis of burnout and gender

#### 3.4.1 Chi-square test

The chi-square test was utilized to determine if there was a significant association between burnout and gender. The resulting p-value of 0.592 suggested that there was no statistically significant relationship between these variables. This implies that the distribution of burnout levels does not differ significantly between male and female students (**Table 2**).

#### 3.4.2 Mann–Whitney U test

As it seemed interesting to us to investigate potential differences in burnout levels between male and female students, the Mann**–**Whitney U test, a non-parametric method for comparing differences between two independent groups, was conducted. The resulting U-value of 17,993.500, with a p-value of 0.554, suggested that there was no significant difference in the median burnout levels between male and female students.

### 3.5 Questions/items worth mentioning

The items mentioned below showed interesting results in the 7-scale score evaluation of the questionnaire based on the answer frequencies, and they are highlighted to focus the attention of the scientific community on the remarkable deviations of perspective that may persist within the student population. Despite the fact that the selective report of the items was not based on a specific statistical tool or measurement, it was the distribution of the answers that made them worth mentioning (**Table 3**). These items shed light on the critical need for awareness of issues such as academic burnout and the challenges students face in maintaining their wellbeing amidst rigorous demands.

**Table 3:** Questions/items worth mentioning of the MBI.

SECTION A Exhaustion: “I feel like my daily life at the University/work is breaking me down.”						
0-Never	1-A few times /year	2-Once a month	3-A few times/month	4-Once a week	5-A few times/week	6-Everyday
0	13	62	78	83	90	74

SECTION B Depersonalization: “I feel tired when I get up in the morning and have to face another day of tasks.”						
0-Never	1-A few times /year	2-Once a month	3-A few times/month	4-Once a week	5-A few times/week	6-Everyday
0	12	49	72	76	88	103

SECTION C Personal Accomplishments: “I feel full of energy.”						
0-Never	1-A few times /year	2-Once a month	3-A few times/month	4-Once a week	5-A few times/week	6-Everyday
0	49	127	124	73	19	8

**Item in SECTION A-Exhaustion: *“I feel like my daily life at the University/work is breaking me down”.***

Mean score ( $\bar{x}$ = 3.94). Taking into consideration that this item is indicative of burnout, it was observed that over 61% scored at least 4 points (i.e., once a week) or more, which indicates that many students feel affected and believe that their university life is draining their daily energy.

**Item in SECTION B-Depersonalization: *“I feel tired when I get up in the morning and have to face another day of tasks”.***

Mean score ( $\bar{x}$ = 4.22). The analysis of this item and distribution of answers revealed that almost half of the participants (n= 191) scored at least 5 points (i.e., a few times per week) or more in the questionnaire. Thus, it is worth questioning whether the population feels some level of difficulty in managing their daily responsibilities.

**Item in SECTION C-Personal Accomplishment:* “I feel full of energy”.***

Mean score ( $\bar{x}$ = 2.78) This question deserves attention as it shows that most respondents felt they lack energy, with precisely 75% (n=300) scoring at least 3 points (i.e., a few times per month) or less, feeling quite drained in this regard while less than 1% reported feeling excessively energetic (i.e., 6 points/every day).

## 4 Discussion

Prior research of international student populations demonstrates that burnout is a universal public health matter, affecting all medical students around the globe, despite their religion, cultural standards, or geographical location. From US to Chile and Spain, studies reveal that no less than half of all medical students risk suffering from burnout (21, 22), with women being more at risk than men (11, 23, 24). However, our study revealed that gender does not play a major role in the burnout distribution. In addition, previous studies revealed that increasing years of study seem to be a risk factor for burnout among medical students (18, 25). This is in accordance with our study, which indicated that students in higher years of study may experience slightly higher levels of burnout (11, 26, 27).

We should not forget that burnout may seem to be an innocent health matter, yet it hides serious dangers for the students’ mental health, such as depression, anxiety, substance abuse, sleep disorders, and even suicidality (28, 29). According to a meta-analysis of over 4,000 articles, vulnerability to burnout increases the risk of suicidality by a factor of six. In addition, burnout is associated with numerous negative physical health outcomes, such as coronary heart disease, musculoskeletal pain, and type 2 diabetes (30).

Preventive measures against the syndrome should be applied, with the most highly recommended being cognitive-behavioral therapy (CBT) and mindfulness, which, according to studies, result in decreased circulating levels of C-reactive protein and proinflammatory cytokines, increased telomere length and telomerase activity, and reduced proinflammatory cytokines in those with depression and anxiety. In medical students specifically, stress-related epigenetic expression of SLC6A4, a serotonin transporter gene, has been found to increase after mindfulness interventions while serum cortisol decreased (31).

Over and above that, the academic system in medical schools worldwide, including Greece, should be shaped with this syndrome in mind, as education providers should question whether they are, in reality, creating undergraduate mental health patients instead of healthy doctors. In this direction, curricula should emphasize the inclusion of good quality sleep, physical activity, and extracurricular activities in the everyday routine of students, as their protective role has been repeatedly proven (32). The integration of optional and time-flexible burnout education into the curriculum would equip students with the fundamental knowledge to identify, prevent, and manage burnout early on (33). Screening tools in the form of questionnaires should always be included during the educational trajectory.

Through the three aspects for evaluating the syndrome, i.e., exhaustion, depersonalization, and low satisfaction for self-achievement, it has been observed that the latter may be very protective against the illness. Undergraduates who achieve a high academic performance and, in general, high personal accomplishment, are more protected from the syndrome. As a result, encouraging students to achieve better academic performance despite mental fatigue would also be a protective strategy against the syndrome (21). Moreover, another invaluable solution would be the adult learning theory, consisting of a six-step procedure of education between the learner and the teacher. The procedure tries to organize the plan of the former and seems to play a very protective role, mainly based on the fact that learners should always seek pure desire-focused personal knowledge enrichment (34).

In conclusion, the syndrome’s prevalence, as shown by the survey, is scientifically important, urging the academic community to examine whether, instead of shaping healthy doctors, the academic system in reality produces patients with the syndrome. To address this condition, academics in medical schools should establish greater awareness and understanding of burnout and of the factors that lead to its development. Interventions focusing on generating wellness during medical studies, such as CBT, mindfulness, the six-stage adult learning technique, periodical screening of the syndrome, and encouragement for higher personal achievements, are highly recommended.

## 5 Strengths and limitations of our study

Up to now, there are no available studies of the burnout syndrome among medical students in Greece, and as far as we know, this was the first study of its kind.

Yet, the present study had some limitations: i) the lack of many other covariates of interest besides gender and year of study; ii) the impact of the previous lockdown on students’ health and education; iii) the results based on self-report information leading to potential bias; iv) online surveys are subject to criticism regarding data quality; v) the so-called “volunteer-effect” of online surveys, in which respondents participate to surveys when they are particularly interested in the topic or when they identify themselves with the survey’s scope. Therefore, bias linked with self-selection should be taken into consideration, since responders’ characteristics may differ substantially from non-responders, limiting the results’ generalizability.

## Data Availability

The original contributions presented in the study are included in the article/supplementary material. Further inquiries can be directed to the corresponding author.
